# Running a dense air temperature measurement field campaign at the urban neighbourhood level: Protocol and lessons learned

**DOI:** 10.1016/j.mex.2022.101719

**Published:** 2022-05-02

**Authors:** Roland Kraemer, Paul Remmler, Jan Bumberger, Nadja Kabisch

**Affiliations:** a Geography Department, Humboldt-Universität zu Berlin, Berlin, Germany; b Department of Monitoring and Exploration Technologies, Helmholtz Centre for Environmental Research – UFZ, Leipzig, Germany; c Department of Urban and Environmental Sociology, Helmholtz Centre for Environmental Research – UFZ, Leipzig, Germany; d Institute of Physical Geography and Landscape Ecology, Leibniz University Hannover, Germany

**Keywords:** Sensors, Urban green spaces, Ecosystem services, Microclimate regulation, Urban climate, Heat, Leipzig

## Abstract

The cooling capacity of urban green spaces constitutes a key measure for cities to mitigate heat events, which is gaining importance in climate change adaptation and mitigation. In this protocol article, we present details on two field campaigns aiming at collecting dense air temperature data in two urban inner city parks in Leipzig, Germany, under unprecedented heat and drought conditions. We introduce all the steps required to plan and conduct qualified fieldwork in environmental research, including study design, technical and logistical preparations, on-site work and data management steps from data acquisition, transfer into research outcomes to dissemination. We further share valuable lessons learned before, during and after fieldwork that helped us improve our work and that could support and improve similar future project campaigns.

## Graphical abstract

**Figure:**
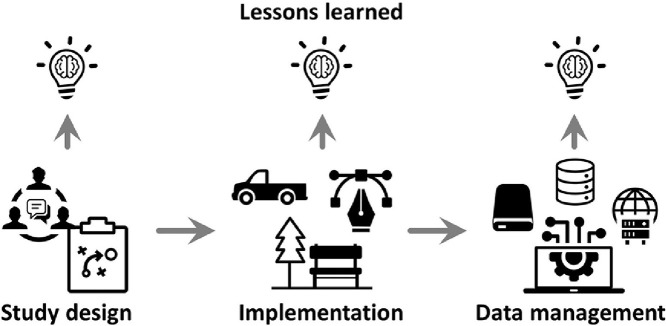

Specifications table

**Table:** 

Subject Area:	Environmental Science
More specific subject area:	*Urban Geography, Ecosystem Services*
Protocol name:	*Meteorological measurements*
Reagents/tools:	Sensors, loggers and other electrical devices HOBO U23–002 Data Logger by Onset HOBO UTBI-001 Data Logger by Onset HOBO Optic USB Base Station by Onset **Further equipment:** See Supplementary Material
Experimental design:	*Temperature survey in public urban spaces*
Trial registration:	*NA*
Ethics:	*NA*
Value of the Protocol:	*Detailed protocol covering all steps required to plan and conduct high-quality fieldwork in environmental research* *Insights for developing individual equipment and into data management* *Lessons learned to facilitate future environmental research in a public urban setting*

## Description of the protocol

### Background

Urban green spaces such as public parks provide a number of ecosystem services that help to address challenges related to climate change and urbanization [1,2]. Vegetation regulates the microclimate through shading and transpiration, and green spaces promote physical activity, relaxation and social interaction and thus are beneficial for human health and wellbeing [3,4]. The potential to provide these ecosystem services may be impaired under extreme weather events such as heat waves and droughts. To assess the capacity of urban parks to provide ecosystem services under heat and drought conditions, namely, to regulate air temperature, we conducted two measurement campaigns during intensive summer heat and drought periods in 2018 and 2019 in two inner-city parks in the city of Leipzig, Germany [5].

To date, to our knowledge no high-quality standard recommendations and guidelines for similar measurement campaigns are available. In this protocol, we provide detailed information on the adopted approach of stationary air temperature measurements for assessing the cooling function of park vegetation under challenging urban heat and drought conditions. Our protocol covers all steps required to plan and conduct high-quality fieldwork in environmental research, including information regarding (i) the study design and fieldwork preparation, (ii) on-site work, and (iii) data management following FAIR guidelines (cf. [6,7]), i.e., making data findable, accessible, interoperable and reusable. Notably, we transparently describe our approach, show some data analysis results as research outcomes and provide lessons learned from our campaigns regarding each of the three steps. Hereby, we hope to improve and facilitate future environmental measurement campaigns, particularly to research the impacts of climate change.

### Study design and fieldwork preparation

The two measurement campaigns were part of a larger project effort (www.greenequityhealth.hu-berlin.de) and focused on two structurally distinct inner-city parks in the city of Leipzig, Germany (e.g., [3,8]). We started initial preparations of these campaigns in spring 2018, roughly four months in advance of the first campaign. The preparation steps covered the following aspects:

• Definition of the study design including the particular sites of interest and period (What? When? Where? How?),
• Selection and acquisition of suitable sensors,
• Development and acquisition of the required equipment,
• Acquisition of official authorization from city administration and potential land owners, and
• Planning of personnel and logistics.

In the following, the preparation steps are explained in greater detail.

#### Study design

**Fig. 1:**
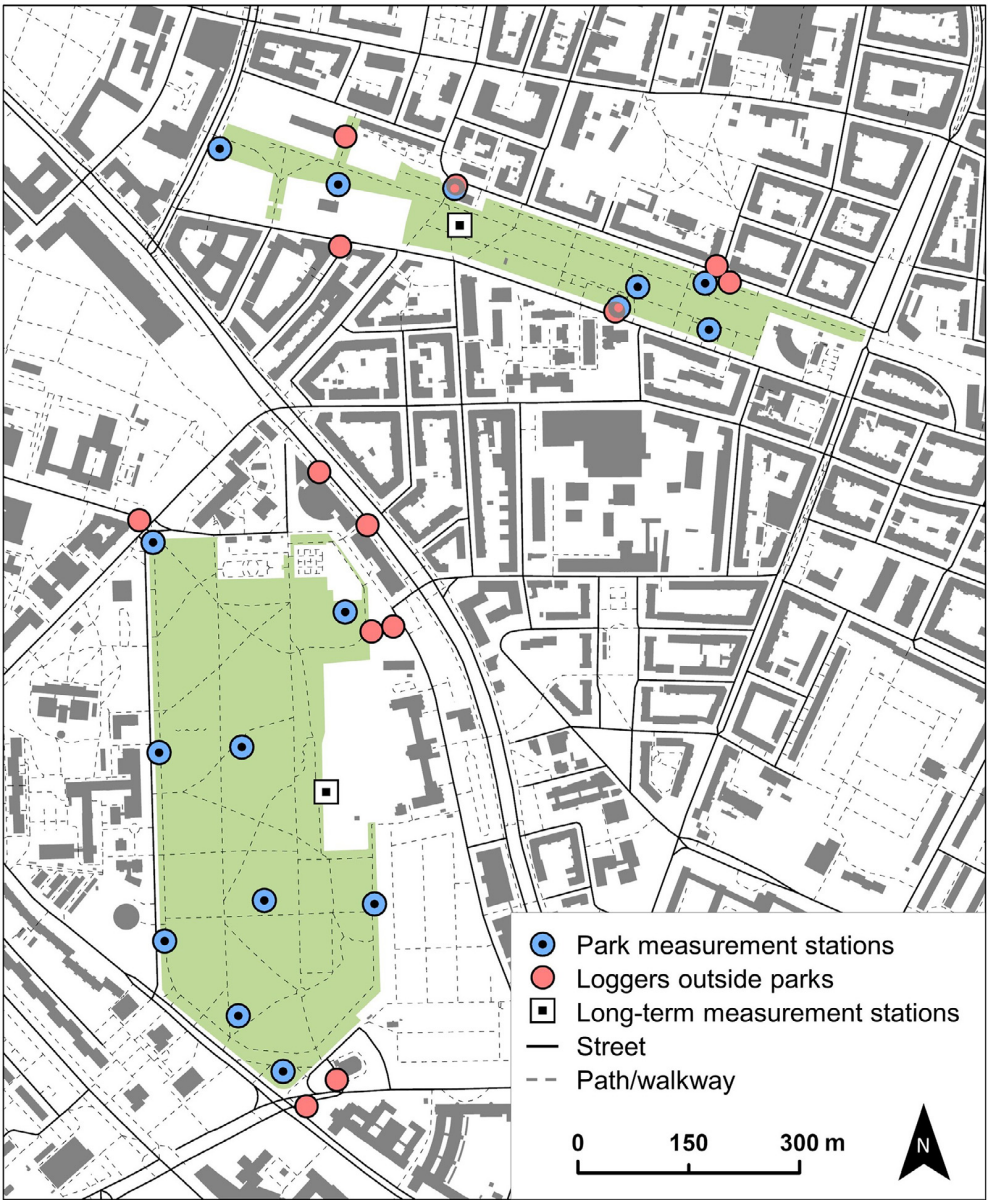
Study area in the city of Leipzig defined by the two major parks, Friedenspark and Lene-Voigt-Park (in green), and their vicinity with locations of air temperature measurements.For more details on local conditions and the location in the city please refer to [9,10]

Based on the project goals, our field campaigns aimed to measure air temperatures at a fine spatial scale in the two parks, Friedenspark (17.5 ha) and Lene Voigt Park (5.8 ha), in the southeastern part of Leipzig (Fig. 1). Prior to our field campaigns, in April 2018, we visited the study sites to explore suitable spots for air temperature measurements. Gaining information about site conditions included information about the local park structure in terms of terrain, park infrastructure and facilities as well as tree coverage that we used to decide for measurement locations.

To efficiently measure the air temperature (by using available capacities), we selected measuring locations based on spatial representativity, i.e., covering the structural diversity of these parks [9], such as park boundaries and densely vegetated and relatively open spaces (for a detailed and interactive view on both parks please refer to [10] and may change the base map). Both parks have flat terrain, except for a small hill in the Friedenspark, and no water bodies such as streams, ponds or lakes. Moreover, we considered our campaign and measurements to least likely interfere with park users and their activities. Based on these requirements, we identified nine locations in the Friedenspark and seven locations in the smaller Lene Voigt Park. This apparent imbalance in measurement point density between the two parks is due to the higher complexity of the shape and internal structure of the Lene Voigt Park, which we intended to capture with a higher measurement point density.

Complementary to the dense measurements during our summer field campaigns, to record long-term data in these parks, we also planned two permanent measurement stations for the entire project duration (2018–2022). For this reason, we identified suitable fenced areas in or at least very close to these parks. During the aforementioned site visits, we also carried out test drillings to determine soil properties and to better understand biophysical conditions in preparation for the actual campaign. We assessed soil composition, depth and porosity directly on site to gain a better understanding of the conditions for vegetation growth and vitality and for water retention (for details please refer to the supplementary material for [5]).

To compare air temperatures within these parks to air temperatures in built-up reference areas, we also designed a sampling strategy outside the two parks, i.e., mainly in surrounding street areas. At this, we focussed on the direct park surroundings to capture the influence of both parks on air temperatures at close range, i.e., within a distance of about 50 m. Locations further away might be under stronger influence of other green spaces which can be found in large numbers in the study area (cf. [4,10]). As with the measurements in the parks, we determined final sensor locations based on site conditions, resulting in a total of 13 off-park locations, as shown in Fig. 1. For reasons of standardization and robustness of data (cf. [11] regarding lack of standardization in urban microclimate studies), we chose stationary measurements at a 2-m height, which is in line with national and international standards [12,13]. Details of the study design are summarized in Table 1.

**Table 1:** Key points of the study design.

Area covered	Measured variable	Time period	Number of measuring points
Ca. 25 ha (two parks of 17.5 and 5.8 ha plus the direct vicinity)	Air temperature at a 2-m height	1 week during the Central European summer (June/July) in 2018 and 2019, respectively	16 within the parks complemented by two long-term measurement stations; 13 outside the parks/in street areas

We aimed to measure the air temperature over a period of approximately one week under summer heat conditions. Accordingly, we prepared for a field campaign in the months of June and July when the annual air temperature normally peaks in our study region. We decided on the actual period for our campaign approximately one week in advance based on weather forecasts.

When planning the schedule of our summer campaigns, we also considered acquisition dates of relevant earth observation satellites to obtain spatially explicit reference data that may allow us to run a citywide assessment of air temperatures using our ground measurements as training data (cf. [14]). Hereby, we focused on the Landsat 8 mission, which also carries a thermal infrared sensor (TIRS) with a spatial resolution of 100 m allowing to derive land surface temperatures (the acquisition dates are available at https://landsat.usgs.gov/landsat_acq).

#### Selection of suitable sensors and loggers

We selected our sensors and data loggers based on the following criteria:

• designed for outdoor environments (waterproof),
• suitable for public surveys (reasonable in size, unobtrusive),
• designed for autonomous data logging, i.e., energy supply for at least one week,
• facilitate fast and easy data transfer,
• mountable with solar radiation shields, and
• provide a reasonable accuracy (< ± 0.25 °C).

**Fig. 2:**
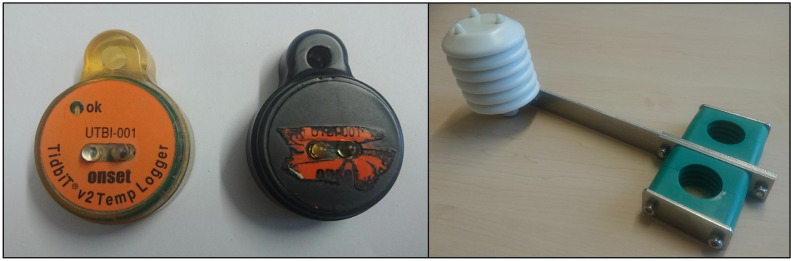
HOBO UTBI-001 data logger as acquired from the manufacturer and coated with black spray paint (left) and logger bracket for the HOBO U23–002 data logger, including radiation shield.

Based on these criteria, we chose the HOBO U23–002 data logger (by Onset Computer Corp.) with an external temperature and relative humidity sensor (link to the datasheet: https://www.onsetcomp.com/datasheet/U23–002). In addition to meeting our criteria, U23–002 data loggers allow users to set up individual logging times and intervals as well as time stamp formats. Regarding the rather exposed measurements in street areas, we chose the smaller and robust HOBO UTBI-001 data logger (by Onset Computer Corp.) with an integrated temperature sensor (link to the datasheet: https://www.onsetcomp.com/datasheet/UTBI-001). To reduce the conspicuousness of the logger as much as possible and to mask the regularly blinking status of the LED, particularly during the night-time, we coated the otherwise transparent casing of the logger with black spray paint (Fig. 2 left). Both loggers were set up and read by a USB base station via an optical interface (HOBO Optic USB Base Station by Onset).

#### Development and acquisition of the required equipment

Regarding the U23–002 data loggers, we commissioned a solar radiation shield for the external sensor combined with a bracket for the logger device (Fig. 2 right). The radiation shield consists of three disc types: one for the top and the bottom, respectively, and four discs of another type in between. We provide a full list of technical drawings for all components in the Supplementary Material. We mounted the U23–002 loggers on the brackets in the lab prior to the fieldwork.

Regarding in-park installation of the sensors, we employed stands specifically designed to be robust, easy to install, as inconspicuous as possible, easy to remove from the site, and ideally reusable. Each of these measuring stands consisted of a stainless steel mounting rod and a ground sleeve as a bracket. To facilitate the installation of the ground brackets, we commissioned an impact rod from our in-house workshop (refer to the technical drawings in the Supplementary Material).

#### Authorization acquisition for the field campaigns

To facilitate our fieldwork in the chosen public parks, we requested authorization from city administration to implement the designed campaigns in publicly owned areas. We contacted the Department for Urban Green and Water of the city of Leipzig in spring 2018 to introduce our project goals and to request permission to conduct our fieldwork. Regarding our long-term measurement stations, we also required areas within or next to the parks that were fenced and hence protected against vandalism. In both parks, we managed to gain access to protected sites in public ownership throughout the entire project duration.

#### Planning of personnel and logistics

The preparation and execution of our field campaigns required the equivalent of one scientific employee (full-time position) supported by a technician who assisted with devices and data management. Regarding the fieldwork, we additionally hired one student assistant. We also ensured that over the period of our fieldwork (June/July), necessary transportation vehicles would be available to transport all equipment to the sites and provide adequate transportation assistance during fieldwork in these parks. For this reason, we reserved suitable vehicles at our research institute three months in advance and ensured that staff with the necessary permission to drive these particular vehicles were available.

Regarding the campaign, we also implemented all the necessary occupational health and safety measures. This included the provision of first aid materials, sun protection and a list of emergency doctors. We further ensured that a trained first aid provider was present at all times during the campaign.

#### Lessons learned from study design and preparation

I. Most importantly, detailed local knowledge about site conditions is necessary when designing a study and planning fieldwork in environmental science. Even though we initially planned our campaigns virtually using maps and high-resolution imagery, our preparatory site visits were essential to identify suitable measuring locations, e.g., identify fenced areas and make decisions on the exact locations of the sensors, and to be suitably prepared for the required practical on-site work, e.g., regarding tools necessary to manage soil conditions.
II. We learned that approaching officials and identifying the right contact persons to request authorization takes time. The response times can be significantly longer than what is expected from colleagues in academia. Hence, several weeks should be planned in a project for these enquiries. It was particularly complex to identify the rightful owners of the selected fenced sites for our long-term stations. Nonetheless, establishing contacts with officials and local stakeholders also provides benefits in the longer term, e.g., in terms of communicating and disseminating project results or partnering in follow-up projects.
III. When planning a weather-dependent campaign, the project team must be flexible. As we aimed to run our campaign during a hot (and dry) summer period, we needed to react flexibly to relatively short-term weather forecasts, i.e., two to three days in advance. Therefore, we scheduled the necessary staff and logistic equipment for a longer period than the actual duration of the campaign. In our case, aiming at a 7-day campaign, we set up a schedule of four weeks. At this point, it is important to communicate well in advance with all people involved that fieldwork may also require working on weekends.

### On-site work

We conducted our first field campaign from July 19 to 26 in 2018 and repeated the campaign in 2019 from June 29 to July 3 with certain modifications and improvements, which we highlight in the Lessons Learned section below.

#### Logistics and equipment

**Fig. 3:**
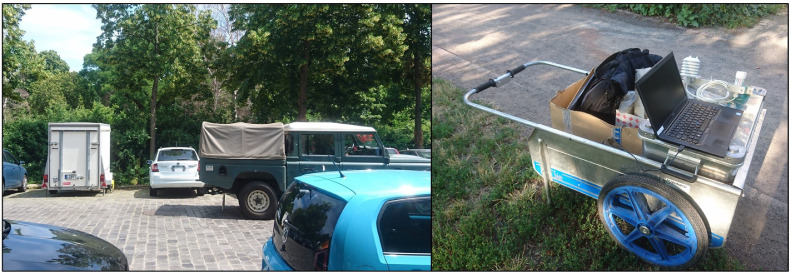
Trailer on a public, unrestricted parking lot next to the Friedenspark (left) and foldable hand cart (right).

To transport all the equipment to the study area, we employed a car trailer. The trailer´s main function, however, was to store our equipment (tools and spare parts) in the study area throughout the entire duration of the campaign. For that reason, we parked the trailer on a public, unrestricted parking lot near Friedenspark (Fig. 3, left). In Lene Voigt Park, we used a fenced area owned by the city to store our equipment during the campaign. Our equipment comprised all necessary parts for the 16 planned measuring stands in the parks (rods and sensor brackets, including two spare parts each) as well as tools to facilitate the on-site work. Regarding transportation of the equipment within the parks, we employed a foldable hand cart (Fig. 3, right). All in all, we had the following equipment for the fieldwork: first aid kit, handcart, impact aids (rod), ground drill, gloves, hex key, open-ended spanner, combination pliers, sunscreen, transport boxes, sledgehammer (small and large), cable ties, folding rule, stepladder, waterproof pen, information sheets.

#### Installation

**Fig. 4:**
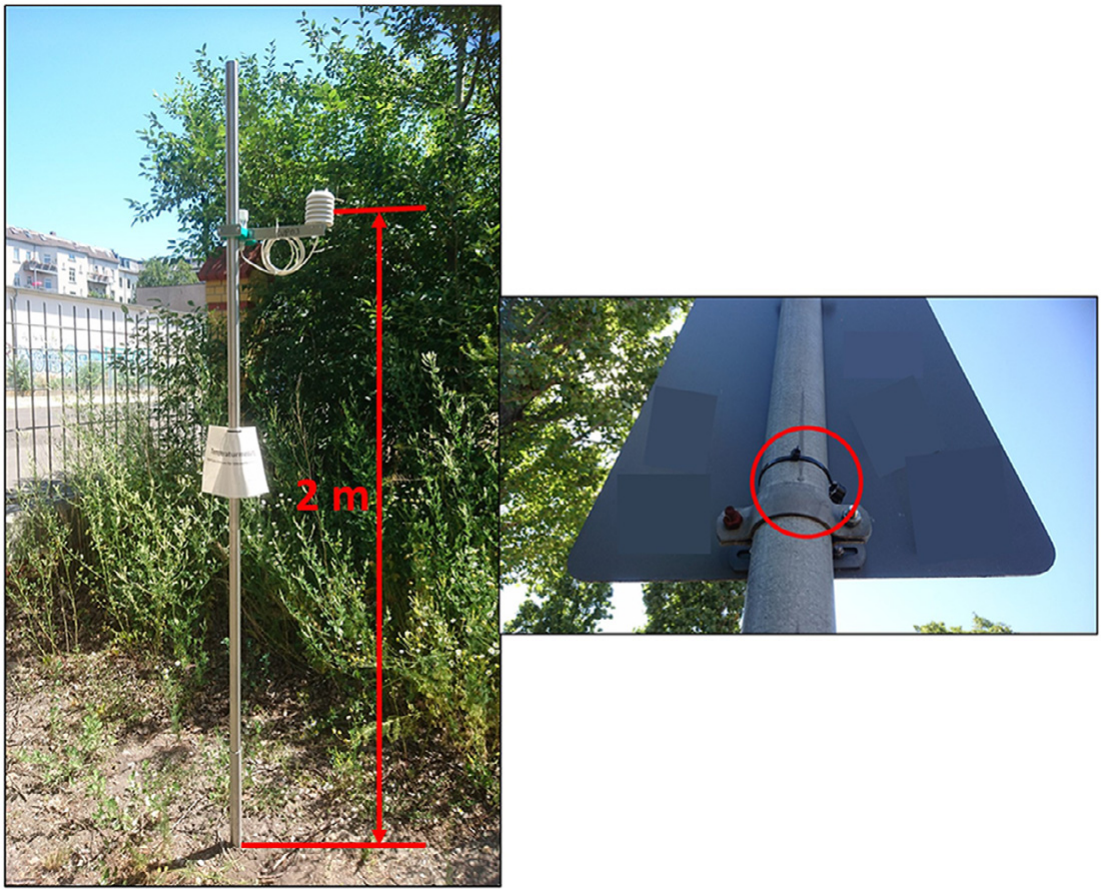
Measuring stands for the U23–002 loggers with information sheets (left) and unshielded UTBI-001 data loggers in nearby street areas attached to traffic signs (right).

The installation of the measuring stands for the U23–002 loggers within the parks (Fig. 4, left) included the following main working steps:

• Driving the bracket/sleeve into the ground with an impact rod and plastic sledge hammer,
• Setting up the mounting rod,
• Mounting the sensor and logger bracket to the rod and adjusting the height to 2 metres,
• Marking the logger brackets with a location ID using a permanent marker, and
• Recording the installation time in the field book for later interpretation of the data.

Recording of the installation time was necessary, because we set up all loggers before we transported them to the sites. In doing so, we chose a 15 min logging interval and synchronized all logging times on the hour. To inform park visitors regarding the purpose of the stands and to provide contact information, we also attached an information sheet to the mounting rods (Fig. 4, left), which we printed and laminated in advance. Comprehensively considered, the setup required approximately 15 to 30 min per site depending on the soil texture/hardness. Combined with the distance travelled between the individual sites, the installation process required three to four hours per park.

We used cable ties to attach the unshielded UTBI-001 data loggers in nearby street areas to traffic signs or wooden planks supporting young street trees (Fig. 4, right). For this, a stepladder was necessary to properly attach the sensors at a height of 2 m. Since the UTBI loggers were not shielded (to keep them as small and inconspicuous as possible), we considered locations shaded by trees or buildings. However, to cover the variety of built-up areas, we explicitly also chose fully sun-exposed locations next to major streets/junctions. The loggers at these exposed locations provided valuable data during the night-time. The setup of all 13 off-park loggers required approximately two hours with a bicycle to cover the distances in between the sites. As for the loggers in the parks, we have set a logging interval for the UTBI-001 loggers of 15 min synchronized on the hour.

We recorded the GPS position of all loggers with a differential GPS and validated the positions using high-resolution aerial images.

#### Operation, maintenance, and dismantling

Our measuring stands within the parks were specifically designed to be easily removable from the public sites. During our first campaign, in fact, we removed exposed/noticeable stands in the evening and set them up again in the early morning to prevent vandalism. To record as much night-time data as possible, however, from day to day we left an increasing number of our sensors in the parks during the night. Eventually, we left all sensors in place during the last two nights of the 2018 campaign and the entire campaign in 2019 with only a few losses due to vandalism. In 2018, two of our measuring stands were damaged and sensors were destroyed (the loggers remained readable), while in 2019, we lost one logger during the night due to vandalism, and one measuring stand was damaged during the daytime.

The risk of vandalism also determined our schedule for logger readout. Accordingly, we backed up data retrieved from our park loggers every evening, which required approximately one hour per park. In addition to the readings in the evenings, we carried out inspection rounds every morning to repair any damage that may have occurred. The small UTBI-001 data loggers in the street areas were left in place 24 h for the entire campaign period, for both years, without any incidents of damage. We retrieved data recorded by the street loggers for the entire measurement period after the campaigns, respectively.

At the end of the first campaign in 2018, we removed all loggers and mounting rods from the park sites but left the brackets in the ground to be reused in the following year. This not only saved a considerable amount of work in 2019 but also ensured identical positions of the measuring stands in both years. After the second campaign in 2019, we also removed the ground brackets from the sites. The small UTBI-001 data loggers were removed after each campaign using a side cutter to cut the cable ties.

#### Lessons learned from the on-site fieldwork

I. One of our most important insights gained during the fieldwork concerns the material of the measuring stands. In the first year in 2018, we used cast metal pods with threaded connections. These cast metal pods, however, were not practical and stable enough for our purpose. The threads were quickly damaged during installation and when we removed and remounted the pods during the campaign. Consequently, in the second campaign in 2019, we developed stands constructed of robust stainless steel that could be simply installed in a ground sleeve and thus easily mountable and removable.
II. Repeating the campaign in 2019 also provided us the opportunity to improve our handling procedures of the sensors and loggers. While we did not pay attention to defined logger times in 2018, i.e., we just initiated logging at 15 min intervals, in 2019, we synchronized the logging times on the hour, which considerably facilitated data analysis.
III. In the course of both campaigns, we experienced less damage to our equipment than expected, particularly when leaving our stands at the sites unattended overnight throughout the 2019 campaign and partly in 2018. The selection of inconspicuous locations for the measuring stands and placement of information sheets probably yielded a positive effect. Notably, the information sheets attached to our stands were indeed useful in multiple ways. We received several messages from interested park visitors asking for more background information, and we were also informed regarding damage to our measuring stands, thus allowing us to react more quickly.

### Data management and outcomes of data analyses

**Fig. 5:**
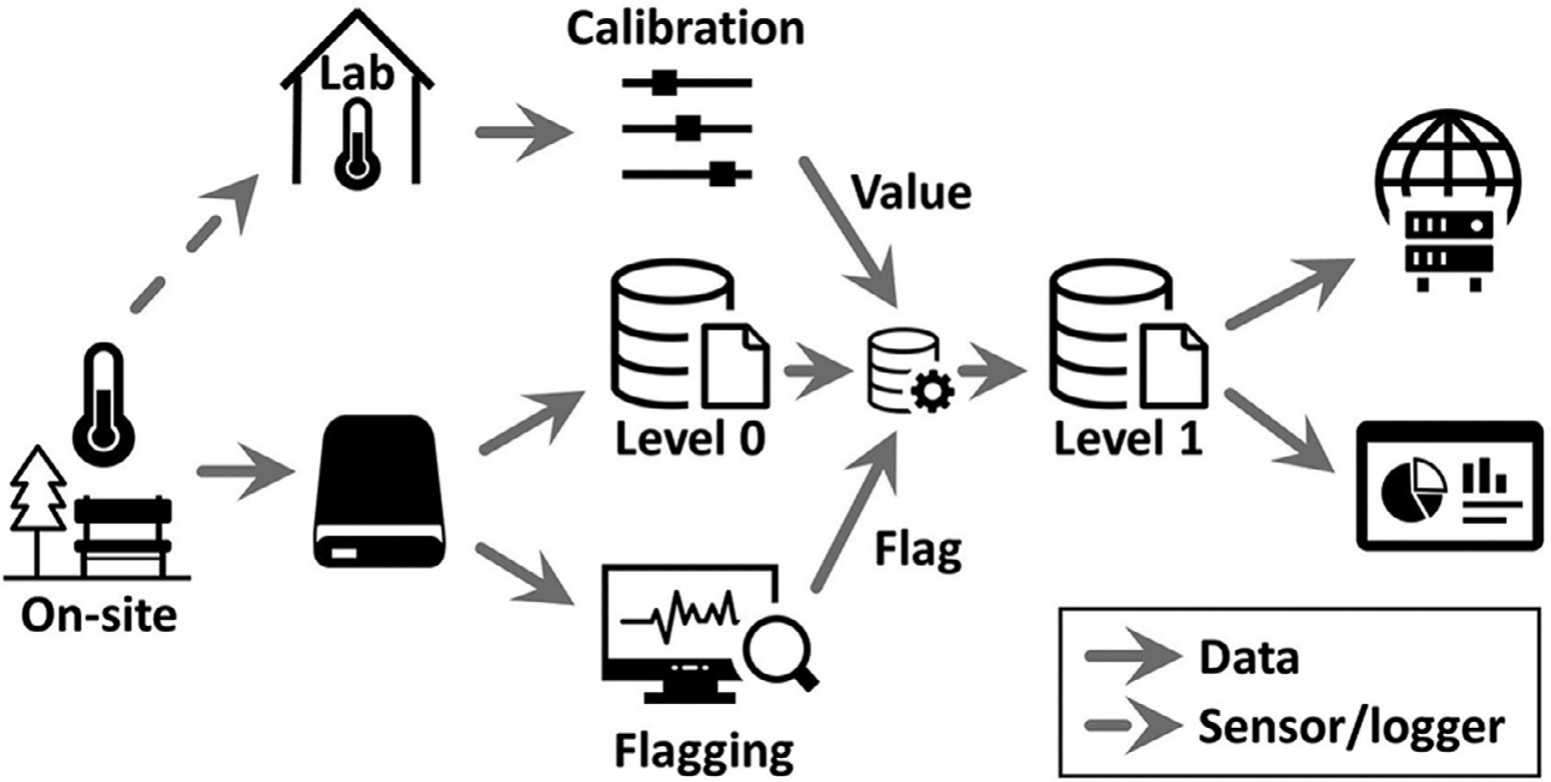
Data flow map.

The processes regarding data acquisition, data processing, data storage and dissemination are shown in Fig. 5. All backup data collected during the campaign were initially stored and compiled on a local physical storage device (hard disk). Once we removed the loggers from the sites, we placed them at one spot in our lab and continued the measurements for approximately 5 days for calibration. With the use of these lab measurements, we determined calibration values for each sensor as the deviation of the sensor mean from the overall mean across all sensors multiplied by −1 (adverse value).

We cleaned the on-site data in terms of potential measurement errors and flagged every single measured value according to campaign events and data plausibility. Values were flagged as suitable (*s*) when the timestamp matched the actual measuring period in the field (i.e., when values were not recorded during transportation) and when the plausibility check was passed. We flagged data as unsuitable (*u*) based on obvious measurement errors as well as data collected during transportation or installation. In particular, we cleaned the data retrieved from the unshielded UTBI-001 loggers considering the radiation effect using our shielded temperature sensors as a reference. We also transferred all raw data to a central server-based database as Level-0 data, i.e., the data stem from the sensors without any processing.

To obtain ready-to-use calibrated and quality-checked data (Level-1 data), we applied the calibration values and flags in the sensor configuration step of our data management system. At this processing step, from Level 0 to Level 1, only values flagged as suitable were passed, and the calibration value was applied. Finally, we made Level-1 data of the 2018 and 2019 campaigns available for download on a public repository [15] and presented the data of our long-term measurement stations via a dashboard on our project website (https://webapp.ufz.de/hubterra/grafana/dashboard/snapshot/tekAkqUu60tXI2K6nvltxQjVoyBzpeXt?orgId=15). Hence, we ensured that our data (including metadata) were findable, accessible, interoperable, and reusable according to FAIR principles [16].

**Fig. 6:**
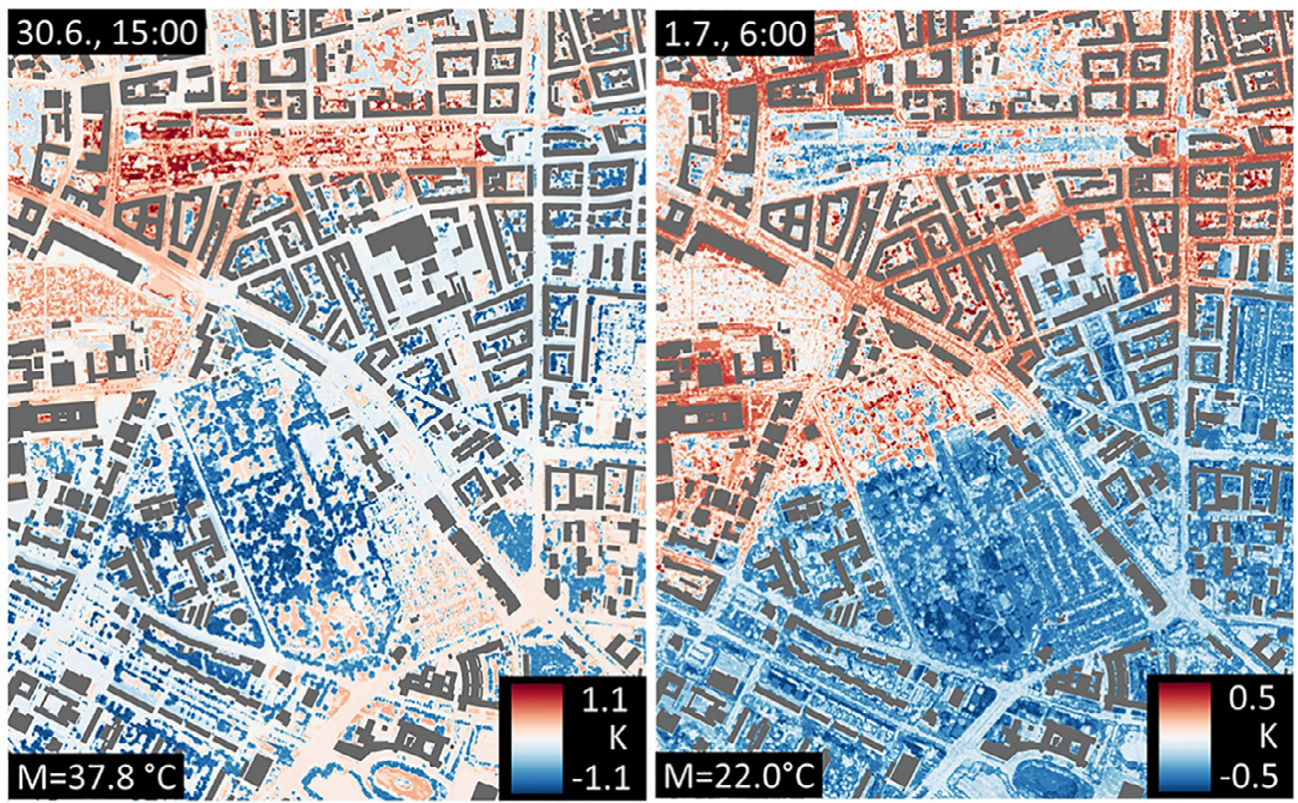
Air temperature deviation from respective mid-range values in Kelvin indicated by M (central value between minimum and maximum, in °C) on 30.6.2019 at 3 pm and on 1.7. at 6 am. Figure adapted from [4].

The data proved suitable for application according to specific research questions. We used the data gained through our temperature measurements in 2018 to study physical activity patterns in parks under conditions of summer heat [3]. In combination with spatially explicit data, e.g., land cover data, we could demonstrate that our dense air temperature measurements in 2019 are suitable to train a machine learning algorithm to obtain spatially explicit predictions of air temperature for a 24 h period [4]. Fig. 6 gives an insight into the results of the study, depicting spatial air temperature patterns at 1 m resolution for two points in time.

#### Lessons learned from data management

I. In our project, we did not establish a data management plan in advance. Instead, we gradually developed our workflow from acquisition to storage, including quality checks, analysis and dissemination using available resources at our institute. This occasionally led to delays in the workflow due to required iterations and improvements. Hence, to operate more efficiently and save resources, we recommend developing a detailed data management plan in advance for any research project faced with data collection. To do so, a few guidelines can be found in the literature, such as those reported by Michener [17], which can be easily transferred to other scientific disciplines.
II. Additionally, related to data management, we learned that sensor calibration should be conducted before installation in the field since sensors can be damaged or even lost during a given campaign. In our case, since we performed calibration after the campaigns, we could not determine a calibration value for three of our sensors that were damaged or lost. In general, due to a technically induced drift in all sensors (in our case 0.1 and 0.01 °C per year), calibration should be timed close to the actual application in the field.
III. By deploying unshielded sensors at the street sites, we originally aimed to collect data for the evaluation of night-time temperatures. Through the selection of shaded locations and thorough cleaning of the obtained data against reference measurements, however, we could also employ some of the data collected during the daytime (cf. [4]).

## Conclusions

In this protocol article, we presented details regarding the preparation, implementation and data management of two field campaigns aimed at collecting dense air temperature data in two distinct urban parks under heat and drought conditions. Given the extensive effort of the project, we learned several lessons that are explained above and again summarized here.

• Site visits are indispensable to obtain information on specific local environmental site conditions to help, e.g., identify suitable measuring locations.
• Obtaining official approval from officials for site use requires time. Officials should thus be approached timely.
• Flexibility in the timing of the actual on-site campaign is needed. Staff and logistic equipment must be organized and be available for a longer period than the actual campaign duration.
• Measurement stands for sensor installation should consist of robust and stable materials (e.g., stainless steel) to ease installation, removal, and potential reuse.
• Logging times should be synchronized across all loggers to facilitate data analysis.
• Information sheets attached to measurement devices with project and contact information were useful to inform park visitors.
• Establishing a data management plan in advance is recommended to avoid delays in the workflow due to required iterations and improvements.
• Sensor calibration should occur before installation in the field.

With these details and our existing published data (open access) according to FAIR principles, we hope to contribute to improved and more highly qualified measurement campaigns in potential future projects aimed at assessing ecosystem service provision under specific environmental conditions. Since urbanization is ongoing [18] and weather extremes are estimated to occur more frequently in the future with severe impacts, particularly in cities [19] where population and values accumulate, interdisciplinary research requires sufficiently detailed measurement outcomes that can help understand the values of urban nature and should also support urban planning for more robust, resilient and sustainable urban development. Most importantly, research and planning efforts are not possible without a team of qualified and motivated staff members, which is the main success factor of fieldwork.
